# Dysregulation of miR-3607 predicts prognosis of hepatocellular carcinoma and regulates tumor cell proliferation, migration and invasion

**DOI:** 10.1186/s13000-020-00973-5

**Published:** 2020-05-13

**Authors:** Wenwen Dou, Min Yang, Yan Su, Ruizhu Xie

**Affiliations:** Department of Infectious Diseases, Affiliated Hospital of Weifang Medical University, No. 2428, Yuhe Road, Kuiwen District, Weifang, 261031 Shandong Province China

**Keywords:** MicroRNA-3607, Tumor progression, Prognosis, Hepatocellular carcinoma

## Abstract

**Background:**

Hepatocellular carcinoma (HCC) is one of the most common global malignancies with increasing morbidity and mortality. The purpose of this study was to investigate the expression levels and prognostic value of microRNA-3607 (miR-3607) in patients with HCC.

**Methods:**

The expression of miR-3607 was estimated by quantitative real-time RT-PCR. Survival analysis using the Kaplan-Meier method and Cox regression analysis was conducted to evaluate the prognostic value of miR-3607. The functional role of miR-3607 in HCC progression was further assessed using gain- and loss-of-function experiments. Bioinformatics analysis and a dual-luciferase reporter assay were used to explore the direct targets of miR-3607.

**Results:**

miR-3607 expression was found to be significantly decreased in HCC tissues and cells compared with the matched tissues and cells (*P* < 0.001). The decreased expression of miR-3607 was associated with the patients’ tumor size and TNM stage (all *P* < 0.05). According to the survival curves, patients with low miR-3607 expression had poorer overall survival than those with high levels (log-rank *P* = 0.012). Moreover, the Cox analysis results indicated that miR-3607 expression was an independent prognostic factor for HCC. The results of cell experiments revealed that the overexpression of miR-3607 in HCC cells led to the inhibited cell proliferation, migration, and invasion. TGFBR1 was identified as a direct target of miR-3607.

**Conclusion:**

The data of this study indicated that the decreased expression of miR-3607 in HCC predicts poor prognosis and the overexpression of miR-3607 in HCC cells can suppress the tumor progression by targeting *TGFBR1*. This study provides a novel insight into the prognosis and treatment of HCC, and miR-3607 serves as a candidate prognostic biomarker and therapeutic target of HCC.

## Introduction

Hepatocellular carcinoma (HCC) is a common global malignancy worldwide and one of the leading causes of cancer-related mortality [1–3]. The statistics indicated that the rates of HCC incidence and mortality are increasing, especially in Africa and Asia [4]. Most of HCC cases are the result of viral hepatitis infections, such as infections of hepatitis B and C [5]. Additionally, the occurrence of HCC is also closely correlated with some metabolic toxins, including aflatoxin and alcohol, and α 1-antitrypsin deficiency and hemochromatosis [6]. Most cases of HCC are determined to be advanced stage tumors at initial diagnosis, which due to the lack of typical symptoms at the early tumor stage of HCC [7]. Although progress has been made in therapeutic strategies, the prognosis of HCC remains not ideal, especially in advanced patients [8]. The high recurrence rate, accounting for approximately 50–70% at 5 years, is still the greatest threat to HCC prognosis [9]. Therefore, there is an urgent need for novel prognostic biomarkers that can help to predict clinical outcomes and direct timely therapy for patients with HCC.

Aberrant microRNAs (miRNAs) have attracted lots of attention for their functional roles during tumor progression and their significant diagnostic and prognostic value in various human cancers [10–13]. miRNAs, a class of small RNAs without protein-coding capacity, can regulate the expression of numerous genes and play pivotal roles during diverse biological processes, such as cell proliferation, differentiation, migration, invasion, and apoptosis [14, 15]. In patients with HCC, some miRNAs with deregulated expression patterns have been determined to be biomarkers for disease diagnosis and prognosis, such as miR-370 [16] and miR-143 [17]. Additionally, some aberrantly expressed miRNAs served as regulator in the progression of HCC and were considered as potential therapeutic targets, such as miR-328-3p [18] and miR-455-5p [19]. The deregulated expression and functional role of miR-3607 in lung cancer [20], colorectal carcinoma [21] and prostate cancer [22] have been reported. For instance, miR-3607 was downregulated in prostate cancer and low miR-3607 expression was correlated with tumor progression and poor survival outcome in prostate cancer patients [22]. Two bioinformatics analysis showed that the expression of miR-3607 was downregulated in HCC samples compared with the normal controls [23, 24]. However, what is the precise expression patterns of miR-3607 and its clinical and functional role in HCC remains unclear.

To uncover the role of miR-3607 in HCC and improve the prognosis and treatment of HCC, this study sought to investigate the expression and clinical significance of miR-3607 in patients with HCC, and explore the functional role of miR-3607 in HCC progression in tumor cells.

## Materials and methods

### Patients and tissues collection

The present study included 122 patients who were pathologically diagnosed with HCC and underwent a surgical operation in the Affiliated Hospital of Weifang Medical University between 2010 and 2013. The patients were recruited based on the following inclusion criteria: (1) all the cases received their first hepatic resection in the Affiliated Hospital of Weifang Medical University, and were pathologically diagnosed as HCC; (2) patients had never received any preoperative antitumor therapy; and (3) clinicopathological data and follow-up information from the patients were completely recorded. The cancerous tissues were collected from these patients who underwent a routine hepatic resection. The adjacent normal tissues were also obtained 3–5 cm from the tumor tissue edge. All of the tissues were immediately frozen in liquid nitrogen after collection and kept at − 80 °C for subsequent RNA extraction. In addition, the clinicopathological characteristics of the patients are summarized in Table 1. The TNM stage of the tumors was determined following the 7th edition of the AJCC cancer staging manual. All of the patients were enrolled in a 5-year follow-up survey, in which the survival information of the patients was recorded for the survival analysis.

**Table 1:** Relationship between miR-3607 expression and clinicopathological data of HCC

Features	No. *n* = 122	miR-3607 expression		*P* values
		Low (*n* = 64)	High (*n* = 58)	
Age (years)				
≤ 50	44	24	20	0.729
> 50	78	40	38	
Gender				
Female	50	26	24	0.933
Male	72	38	34	
Tumor size (cm)				
≤ 5	70	30	40	0.014
> 5	52	34	18	
AFP (μg/L)				
≤ 400	62	30	32	0.360
> 400	60	34	26	
Cirrhosis				
No	47	25	22	0.898
Yes	75	39	36	
TNM stage				
I-II	62	24	38	0.002
III-IV	60	40	20	

*AFP* Alpha Fetal Protein

The experimental procedures of the current study were proved by the Ethics Committee of Affiliated Hospital of Weifang Medical University. The informed consent for the sample collection and application was signed by each participant. The specimens used were all anonymous, following the ethical and legal standards.

### Cell culture and transfection

Four HCC cell lines Huh7, Hep3B, Li7 and SNU449, and a normal hepatocyte cell line L02 were purchased from the Cellcook Cell Biotechnology Co., Ltd. (Guangzhou, China). The cells were cultured in Dulbecco’s Modified Eagle’s medium (DMEM; GIBCO, NY, USA) supplemented with 10% fetal bovine serum (FBS) (GIBCO, NY, USA) and kept at 37 °C in a humidified incubator with 5% CO_2_.

miR-3607 mimic (5′-ACUGUAAACGCUUUCUGAUG-3′), miR-3607 inhibitor (5′-CAUCAGAAAGCGUUUACAGU-3′) and their corresponding negative controls (mimic-NC: 5′-UUCUCCGAACGUGUCACGUTT-3′, and inhibitor-NC: 5′-CAGUACUUUUGUGUAGUACAA-3′) were designed in GeneCopoeia (Guangzhou, China) and transfected into HCC cells at a final concentration of 50 nM using Lipofectamine 2000 reagent (Invitrogen, Burlington, ON, Canada) following the manufacturer’s instructions. miR-3607 mimic was used to upregulate the expression of miR-3607, while the miR-3607 inhibitor was used to downregulate the expression of miR-3607, which were used for gain/loss-of-function experiments. The cells were cultured and collected at 48 h after transfection for use in further cell experiments.

### RNA extraction and quantitative real-time RT-PCR (qRT-PCR)

Total RNAs including miRNAs were extracted from the collected tissues and cells using TRIzol reagent (Invitrogen) as per the manufacturer’s protocols. The reverse transcription was conducted to synthesize cDNA from the RNA with a PrimeScript reverse transcriptase (RT) reagent kit (TaKaRa, Shiga, Japan). In this study, qRT-PCR was performed by using a SYBR Green PCR master mix (Applied Biosystems, USA) and a 7300 Real-Time PCR System (Applied Biosystems, USA) to estimate the expression levels of miR-3607. The reaction was carried out with the following thermocycling conditions: initial denaturation at 95 °C for 3 min, followed by 40 cycles of denaturation at 95 °C for 10 s and annealing at 60 °C for 15 s, then extension at 72 °C for 30 s. The final relative expression of miR-3607 was normalized to *U6* and calculated by the 2^−ΔΔCt^ method.

### Cell proliferation assay

The HCC cells were seeded in a 24-well dish with the cell density of 2 × 10^4^ cells/well and then transfected with the miR-3607 mimic, inhibitor or NCs. 50 μl methyl thiazolyl tetrazolium (MTT) (5 mg/mL) was separately added in each of the well at the time points of 0, 24, 48 and 72 h. The wells were then kept in an incubator at 37 °C with 5% CO_2_ for 4 h. After the incubation, 500 μL 20% SDS was added to the cells and incubated overnight at room temperature. The absorbance of the cells at 490 nm was measured to evaluate the proliferation of the HCC cells.

### Cell migration and invasion assay

In the current study, the HCC cell migration and invasion were assessed using a Transwell analysis with an 8 μm pore size membrane without Matrigel (for migration assessment) or with Matrigel (for invasion analysis). Serum-free medium was added in the top chambers, and the lower chambers were full of medium supplemented with 10% FBS. Cells with a density of 2 × 10^4^ cells/well were seeded into the top of the chambers and incubated for 24 h at 37 °C. The membrane was then fixed with 95% ethanol for 30 min, and the cell staining was performed using 0.2% crystal violet for another 30 min. The cell number was counted using an inverted microscope.

### Bioinformatics analysis and dual-luciferase reporter assay

The potential target genes of miR-3607 were predicted using online publicly software TargetScan 7.2 (www.targetscan.org). To validate whether TGFBR1 was a direct target of miR-3607, the wild or mutated 3’UTR of TGFBR1 was amplified and cloned into the pGL3 luciferase reporter vector (Promega, Madison, WI, USA). Then, wild-type (Wt) or mutated (Mut) luciferase reporter vectors of TGFBR1 were co-transfected with miR-3607 mimic, miR-3607 inhibitor, mimic NC, or inhibitor NC into Huh7 cells, which has better functional results. After 48 h, the relative firefly luciferase activities were measured using dual luciferase assay (Promega, Madison, WI, USA) that normalized to *Renilla* Luciferase activity.

### Statistical analysis

All of the statistical analyses were performed using SPSS 18.0 software (SPSS Inc., Chicago, IL) and GraphPad Prism 5.0 software (GraphPad Software, Inc., USA). Differences between groups were analyzed using Student’s *t*-test or one-way ANOVA followed by Tukey’s multiple comparison test. The Chi-square test was adopted to examine the relationship between miR-3607 expression and the clinicopathological features of HCC. Survival analysis was conducted for the patients using Kaplan-Meier methods and the log-rank test. To confirm the prognostic value of miR-3607, a Cox regression analysis was adopted. A difference with a *P* < 0.05 was considered to be statistically significant.

## Results

### Expression levels of miR-3607 in HCC tissues and cell lines

**Fig. 1:**
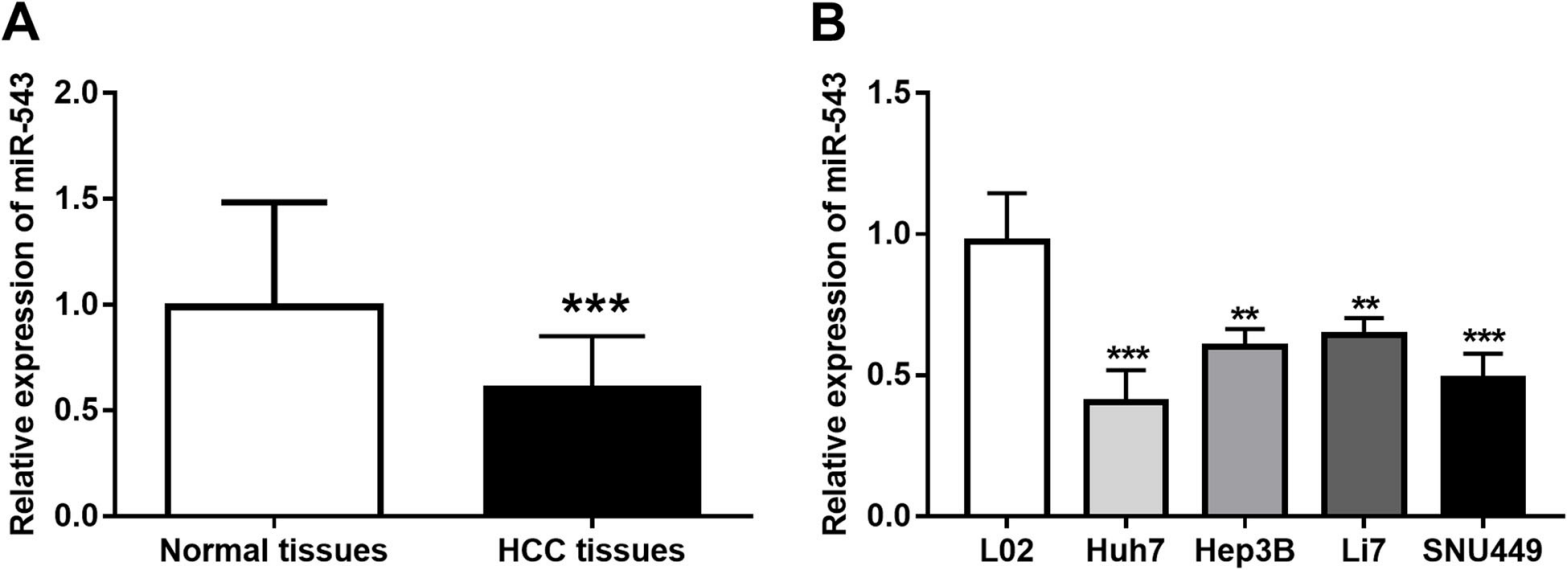
Expression of miR-3607 measured by qRT-PCR in the 122 paired HCC tissues and normal tissues. **a**. miR-3607 expression was significantly downregulated in HCC tissue samples compared with the normal controls (***P < 0.001). **b**. Expression of miR-3607 was decreased in HCC cell lines compared with the normal cells (**P < 0.01, ***P < 0.001)

In the current study, miR-3607 expression levels in the 122 paired HCC tissues and normal tissues were estimated by qRT-PCR. As shown in Fig. 1a, the expression of miR-3607 was markedly lower in HCC tissues than that in the matched noncancerous tissues (*P* < 0.001). The expression results in HCC cells were consistent with the results in tissues, which also showed a significant decrease in the expression of miR-3607 in HCC cell lines (Huh7, Hep3B, Li7, and SNU449) compared with the normal cells (all *P* < 0.01, Fig. 1b).

### Association of miR-3607 expression with the clinicopathological features of patients with HCC

We also focused on the relationship between miR-3607 expression and the patients’ clinicopathological characteristics. Seven parameters were analyzed, including age, gender, tumor size, alpha fetal protein (AFP) concentration, cirrhosis, and TNM stage. The collected patients with HCC were classified into a low miR-3607 expression group and a high miR-3607 expression group based on the mean miR-3607 expression value in HCC tissues. The results of the Chi-square test, as shown in Table 1, revealed that the decreased miR-3607 expression was correlated with larger tumor size (*P* = 0.014) and advanced TNM stage (*P* = 0.002). No statistically significant relationship was found between miR-3607 expression and the other parameters (all *P* > 0.05).

### Prognostic significance of miR-3607 expression in patients with HCC

**Fig. 2:**
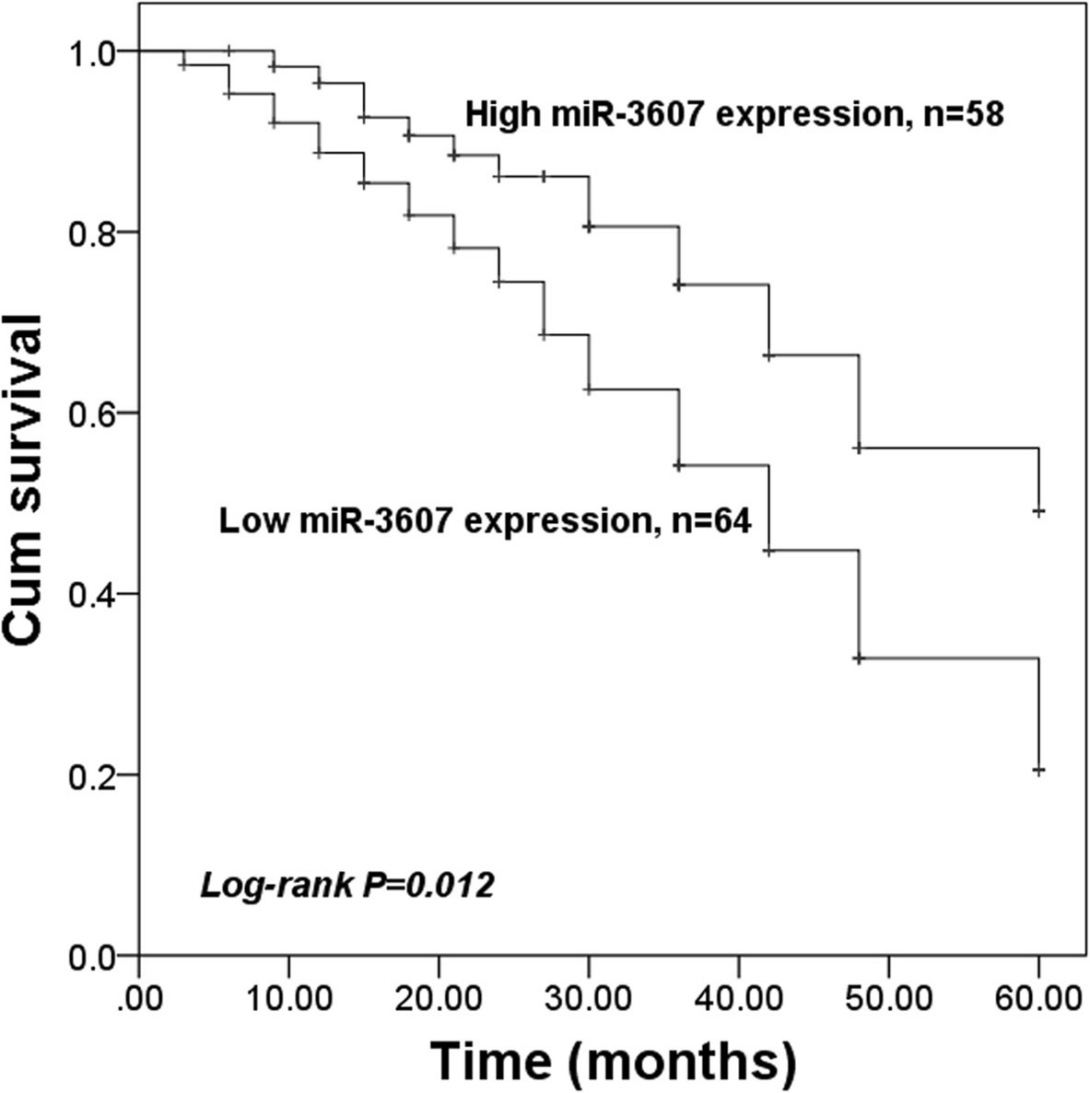
Kaplan-Meier survival analysis for patients with HCC based on the expression of miR-3607. Patients with low miR-3607 expression had poor survival compared to those with high expression (log-rank *P* = 0.012)

To assess the prognostic value of miR-3607 for patients with HCC, a survival analysis, and a Cox regression analysis were performed. From the Kaplan-Meier survival curves shown in Fig. 2, we found that patients with HCC with low miR-3607 expression had poorer survival rates than those with high expression (log-rank *P* = 0.012). Furthermore, miR-3607 and the other clinical parameters, which might be independently correlated with the overall survival of patients with HCC, were all analyzed with Cox analysis. The results of Cox analysis (Table 2) indicated that miR-3607 expression (HR = 2.005, 95% CI = 1.089–3.882, *P* = 0.035) and TNM stage (HR = 1.924, 95% CI = 1.021–3.629, *P* = 0.043) were independent indicators for HCC prognosis.

**Table 2:** Cox regression analysis for miR-3607 in HCC patients

Variables	Univariate analysis			Multivariate analysis		
	HR	95%CI	*P* value	HR	95%CI	*P* value
miR-3607	2.191	1.185–4.052	0.012	2.005	1.089–3.882	0.035
Age	1.139	0.646–2.009	0.652	1.250	0.677–2.309	0.476
Gender	1.286	0.726–2.277	0.389	1.424	0.784–2.585	0.246
Tumor size	1.692	0.977–2.928	0.060	1.459	0.819–2.600	0.200
AFP	1.315	0.751–2.305	0.338	1.304	0.719–2.367	0.382
Cirrhosis	1.349	0.778–2.340	0.286	1.533	0.869–2.703	0.140
TNM stage	2.040	1.132–3.677	0.018	1.924	1.021–3.629	0.043

*AFP* Alpha Fetal Protein, *HR* Hazard Ratio, *CI* Confidence Interval

### Effects of miR-3607 expression on HCC cell proliferation, migration, and invasion

**Fig. 3:**
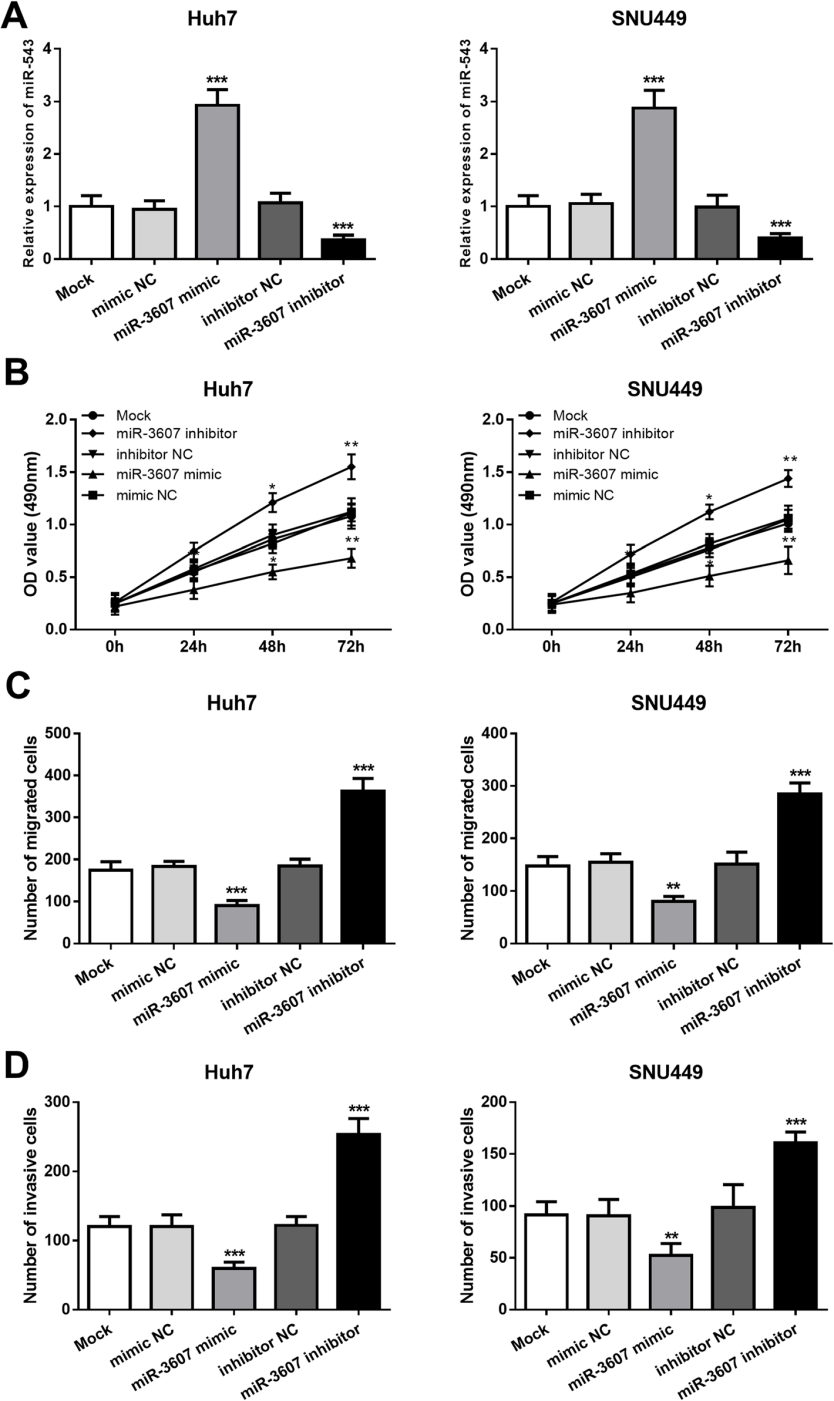
Effects of miR-3607 on cell proliferation, migration and invasion in Huh7 and SNU449 cell lines. **a**. Expression of miR-3607 was upregulated by the miR-3607 mimic and downregulated by the miR-3607 inhibitor (***P < 0.001). **b**. Cell proliferation of Huh7 and SNU449 cells was enhanced by the knockdown of miR-3607, but was inhibited by the overexpression of miR-3607 (*P < 0.05, **P < 0.01). **c** and **d**. The upregulated miR-3607 expression in HCC cells led to suppressed cell migration (**c**) and invasion (**d**), whereas the downregulated miR-3607 expression led to the opposite migration and invasion results (**P < 0.01, ***P < 0.001)

To investigate the effects the functional role of miR-3607 in HCC progression, gain- and loss-of-function experiments were carried out in Huh7 and SNU499 cell lines, which showed the significant low miR-3607 expression levels compared with the normal cell line. The expression of miR-3607 in the two HCC cell lines was significantly upregulated by the miR-3607-mimic and decreased by the miR-3607 inhibitor (all *P* < 0.001, Fig. 3a). According to the MTT assay, we found that the HCC cell proliferation was obviously suppressed by the overexpression of miR-3607, but as enhanced by the downregulation of miR-3607 (all *P* < 0.05, Fig. 3b). For the migration and invasion abilities of Huh7 and SNU499 cells measured by a Transwell assay, the results shown in Fig. 3c and d indicated that the upregulation of miR-3607 in HCC cells led to inhibited cell migration and invasion, while resulted in the opposite migration and invasion results (all *P* < 0.01).

### TGFBR1 was a direct target of miR-3607

**Fig. 4:**
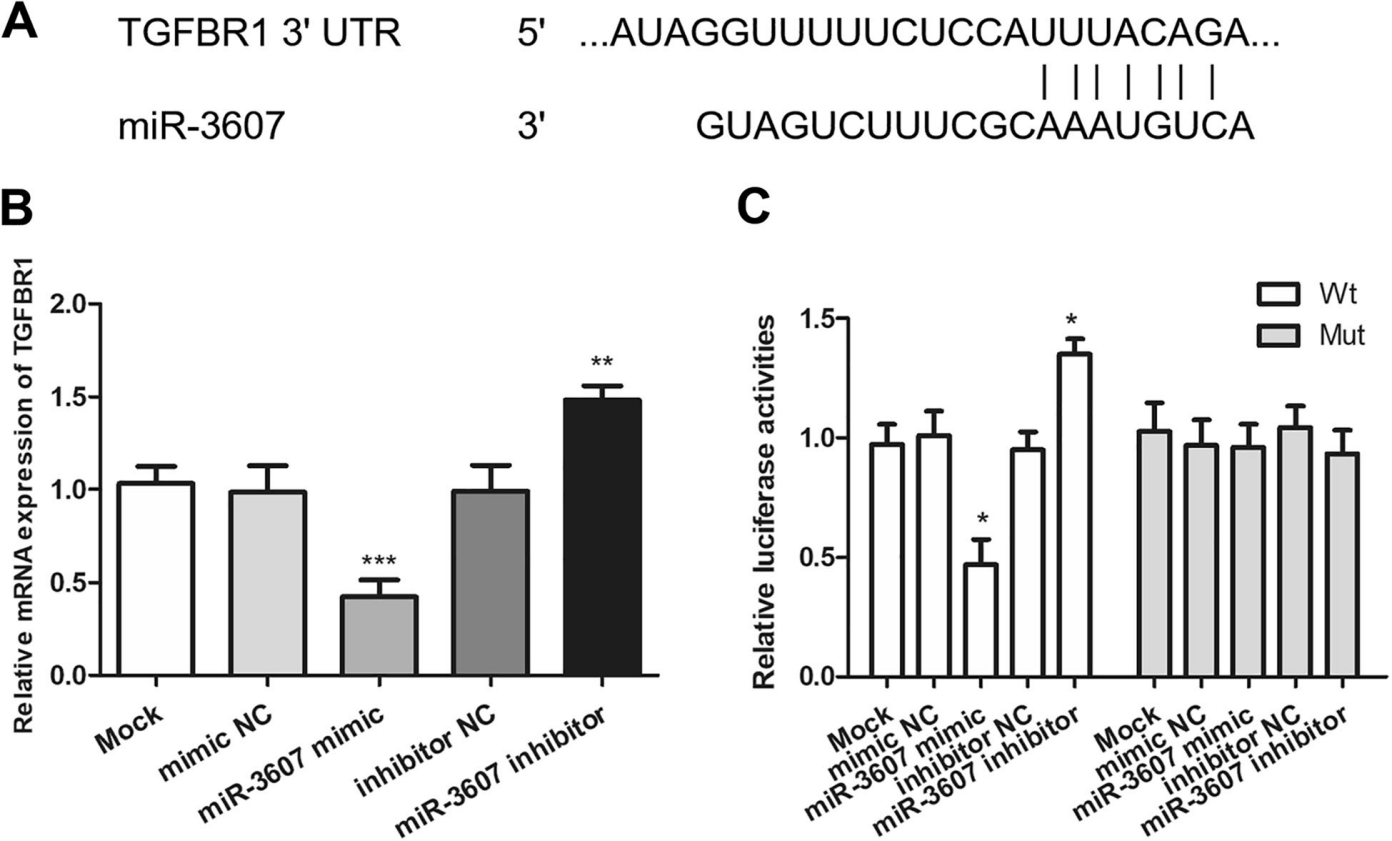
TGFBR1 was a direct target of miR-3607 in HCC. **a**. The putative binding sites of miR-3607 in the TGFBR1 3’UTR. **b**. TGFBR1 expression in Huh7 cells transfected with miR-3607 mimic, mimic NC, miR-3607 inhibitor, or inhibitor NC (**P < 0.01, ***P < 0.001). **c**. Luciferase activities in Huh7 cells co-transfected with luciferase reporters containing TGFBR1-Wt or TGFBR1-Mut and miR-3607 mimics or miR-3607 inhibitors, respectively (*P < 0.05)

The putative potential target genes of miR-3607 were identified by performing an online search of the TargetScan. Among these putative targets, *TGFBR1* was selected for further validation, as it was a widely investigated oncogene in human cancers, including HCC [25]. Data suggested that *TGFBR1* might be a direct target of miR-3607 in HCC (Fig. 4a). Then, we used qRT-PCR assay to measure the mRNA levels of *TGFBR1* in Huh7 cells. As predicted overexpression of miR-3607 markedly reduced the mRNA levels of *TGFBR1*, while knockdown of miR-3607 increased the mRNA levels of *TGFBR1* (*P* < 0.01, Fig. 4b). To confirm the relationship between *TGFBR1* and miR-3607, dual-luciferase reporter assays were carried out and results showed that luciferase activity was markedly decreased in Huh7 cells co-transfected with *TGFBR1*–3’UTR-Wt and miR-3607 mimics (*P* < 0.05, Fig. 4c). However, there was no significant effect on the luciferase activity of Huh7 cells that co-transfected with *TGFBR1*–3’UTR-Mut and miR-3607 mimic. These results confirmed that TGFBR1 was a direct target of miR-3607 in HCC cells.

## Discussion

Accumulated studies report the important roles of miRNA in various human diseases, especially in human cancers. MiRNAs have been reported to be involved in various biological processes with critical regulatory functions [26]. A wide spectrum of oncogenes or tumor suppressors could be regulated by miRNAs, leading to the promotion or suppression of tumor development. For example, the oncogenes of renal cell carcinoma, *ANGPT2*, and *NEDD9*, are the targets of miR-145, which is involved in tumor progression by suppression of the two oncogenes expression [27]. In gastric cancer, miR-223 has been found to downregulate the expression of EPB41L3, which acts as a tumor suppressor of gastric cancer and could promote the cell migration and invasion in gastric cancer cells [28]. These functional miRNAs have aberrant expression patterns in tumor samples and are usually involved in tumor progression. In pancreatic cancer, the expression of miR-494 is downregulated in cancer tissues compared with the paired normal tissues and is correlated with the tumor TNM stage, invasion, metastasis and tumor grade [29]. Inversely, increased expression of miR-215 has been detected in gastric cancer samples and can promote cell migration and invasion in cancer cells [30]. Similarly, some miRNAs with abnormal expression levels have also been investigated in HCC, such as the upregulated miR-191 [31] and the decreased miR-489 [32]. Thus, to examine the expression patterns of miRNAs, as well as their clinical significance and biological function in HCC, is of great importance for the development of cancer treatments.

In the present study, we analyzed the expression of miR-3607 in HCC tissue samples and cell lines using qRT-PCR. The results showed that miR-3607 expression was remarkably lower in HCC tissues and cell lines than that in the corresponding tissue and cell controls, which were consistent with the previously reported expression data of miR-3607 [23, 24], which was demonstrated by the bioinformatics analysis. The data in this study provide clinical and cell experiment evidence for the expression of miR-3607 in HCC. In addition to HCC, the downregulated expression of miR-3607 has also been found in colorectal cancer, breast cancer and non-small cell lung cancer [21, 33, 34]. In another study by Lin and co-workers, miR-3607 was upregulated in lung cancer tissues and cells, and miR-3607 overexpression promoted cell proliferation by suppressing APC [20]. This study results were inconsistent with above other studies, suggesting miR-3607 might play different roles depending on the types of tumor. The further Chi-square test data revealed that the decreased miR-3607 expression was associated with larger tumor size and advanced TNM stage of HCC patients. These above results implied that miR-3607 might be involved in the development of HCC.

Numerous miRNAs have been determined to be noninvasive biomarkers for the diagnosis and prognosis of various human cancers [35]. In patients with HCC, there are also some miRNAs serve as candidate biomarkers. For example, a study conducted by Wu and his colleagues showed that the aberrant expression of microRNA-4651 was observably correlated with cancer diagnosis and prognosis and could serve as a noninvasive biomarker in patients with HCC [36]. MicroRNA-425-5p has also been investigated in HCC tissues and cells and was demonstrated to be involved in tumor progression and to act as a candidate prognostic biomarker in HCC [37]. A previous study by Sharanjot Saini and his colleagues indicated that low miR-3607 expression was correlated with poor survival outcome in prostate cancer [22]. In the present study, the Kaplan-Meier survival curves showed that patients with low miR-3607 expression had poorer overall survival than those with high expression of miR-3607. The further Cox analysis data indicated that miR-3607 expression was an independent prognostic indicator in HCC.

In addition to the clinical value of miR-3607, its functional role in HCC progression was also examined in this study. By using cell transfection, the expression of miR-3607 was successfully regulated in vitro. The overexpression of miR-3607 in HCC cells led to the inhibition in cell proliferation, migration and invasion, whereas the knockdown of miR-3607 resulted in the opposite results, indicating the tumor suppressor role of miR-3607 in HCC progression. In colorectal cancer, miR-3607 was also found to suppress tumor cell proliferation, migration and invasion through targeting DDI2 [21]. In non-small cell lung cancer, the overexpression of miR-3607 in tumor cells could inhibit cell proliferation, migration, invasion and cell cycle by downregulating TGFBR1 and CCNE2 [33]. TGFBR1 is a widely investigated oncogene in human cancers, including HCC [25, 38]. In the present study, TGFBR1 was validated as a novel direct target of miR-3607. Combining with these results, we considered that miR-3607 might inhibit cell proliferation, migration, and invasion by targeting TGFBR1. However, the precise molecular mechanisms of miR-3607 and the role of TGFBR1 and in HCC still need to perform in further investigations. In addition, the sample size in the present study, which may make the clinical results biased. Thus, large scope of patients will also be needed in future researches.

In conclusion, all the data in this study indicated the decreased expression of miR-3607 in HCC patients predicts the poor prognosis and associated with patients’ tumor size and TNM stage. The overexpression of miR-3607 can inhibit HCC cell proliferation, migration and invasion. In addition, TGFBR1 was identified as a direct target of miR-3607. Our results provide a novel insight into the prognosis and treatment of HCC, and the miR-3607/TGFBR1 axis may be a candidate prognostic biomarker and therapeutic target in HCC.

## Data Availability

The datasets used and/or analysed during the current study are available from the corresponding author on reasonable request.

## Abbreviations

HCC: Hepatocellular carcinoma
miR-3607: microRNA-3607
miRNAs: microRNAs
DMEM: Dulbecco’s Modified Eagle’s medium
FBS: Fetal bovine serum
qRT-PCR: Quantitative real-time RT-PCR
RT: Reverse transcriptase
MTT: Methyl thiazolyl tetrazolium
AFP: Alpha fetal protein
